# The Role of HDAC6 in Autophagy and NLRP3 Inflammasome

**DOI:** 10.3389/fimmu.2021.763831

**Published:** 2021-10-27

**Authors:** Panpan Chang, Hao Li, Hui Hu, Yongqing Li, Tianbing Wang

**Affiliations:** ^1^ Trauma Medicine Center, Peking University People’s Hospital, Key Laboratory of Trauma and Neural Regeneration (Peking University), National Center for Trauma Medicine of China, Beijing, China; ^2^ Department of Emergency, First Hospital of China Medical University, Shenyang, China; ^3^ Department of Traumatology, Central Hospital of Chongqing University, Chongqing Emergency Medical Center, Chongqing, China; ^4^ Department of Surgery, University of Michigan, Ann Arbor, MI, United States

**Keywords:** HDAC6, autophagy, NLRP3 inflammasome, inflammation, post-translational modification

## Abstract

Autophagy fights against harmful stimuli and degrades cytosolic macromolecules, organelles, and intracellular pathogens. Autophagy dysfunction is associated with many diseases, including infectious and inflammatory diseases. Recent studies have identified the critical role of the NACHT, LRR, and PYD domain-containing protein 3 (NLRP3) inflammasomes activation in the innate immune system, which mediates the secretion of proinflammatory cytokines IL-1β/IL-18 and cleaves Gasdermin D to induce pyroptosis in response to pathogenic and sterile stimuli. Accumulating evidence has highlighted the crosstalk between autophagy and NLRP3 inflammasome in multifaceted ways to influence host defense and inflammation. However, the underlying mechanisms require further clarification. Histone deacetylase 6 (HDAC6) is a class IIb deacetylase among the 18 mammalian HDACs, which mainly localizes in the cytoplasm. It is involved in two functional deacetylase domains and a ubiquitin-binding zinc finger domain (ZnF-BUZ). Due to its unique structure, HDAC6 regulates various physiological processes, including autophagy and NLRP3 inflammasome, and may play a role in the crosstalk between them. In this review, we provide insight into the mechanisms by which HDAC6 regulates autophagy and NLRP3 inflammasome and we explored the possibility and challenges of HDAC6 in the crosstalk between autophagy and NLRP3 inflammasome. Finally, we discuss HDAC6 inhibitors as a potential therapeutic approach targeting either autophagy or NLRP3 inflammasome as an anti-inflammatory strategy, although further clarification is required regarding their crosstalk.

## Introduction

Autophagy is a conservative mechanism for maintaining homeostasis in cells, which degrades misfolded proteins, damaged organelles, and intracellular pathogens (1). It is associated with many diseases, including infectious and inflammatory diseases (2). The NACHT, LRR, and PYD domain-containing protein 3 (NLRP3) inflammasomes are oligomeric complexes activated by invading pathogens, endogenous danger signals, and stress signals (3). The activation of NLRP3 inflammasome induces interleukin-1β (IL-1β) and interleukin-18 (IL-18) release and pyroptosis, which is a caspase-1-dependent form of programmed cell death (4). NLRP3 inflammasome is essential for defense against infectious and inflammatory diseases, and its aberrant activation aggravates inflammation and tissue damage (5, 6). Recent studies have suggested that autophagy eliminates the overaction of NLRP3 inflammasome and maintains homeostasis (7–9). Additionally, NLRP3 inflammasome activation can upregulate autophagy to suppress excessive responses and protect the host (10, 11). There is emerging evidence highlighting the importance of crosstalk between autophagy and NLRP3 inflammasome in various inflammatory diseases (12–16).

Histone deacetylase 6 (HDAC6) is a class IIb deacetylase found in 18 mammalian HDACs. It harbors two functional deacetylase catalytic domains and a ubiquitin-binding zinc finger domain (ZnF-BUZ) (17). HDAC6 is a structurally and functionally unique cytoplasmic deacetylase that can deacetylate multiple non-histone proteins such as α-tubulin, cortactin, heat shock protein (HSP90), heat shock transcription factor-1 (HSF-1), peroxiredoxin I (Prx I), and peroxiredoxin II (Prx II) (18–21). In addition, HDAC6 binds to ubiquitinated misfolded proteins through the ZnF-BUZ (22). Therefore, it is essential for multiple physiological and pathological processes. Recent studies have demonstrated that HDAC6 regulates autophagy and NLRP3 inflammasome activation through various mechanisms (14, 23–27). It is suggested that HDAC6 plays a possible role in the crosstalk between autophagy and NLRP3 inflammasome, although there is little direct evidence to date. In this review, we present the distinct roles of HDAC6 in the regulation of autophagy and NLRP3 inflammasome. We then focus on exploring the possibility and challenges of HDAC6 involvement in the crosstalk between autophagy and NLRP3 inflammasome. Finally, we discuss HDAC6 inhibitors as a promising therapeutic target for various diseases and its prospect in the crosstalk between autophagy and NLRP3 inflammasome.

## The Role of HDAC6 in Autophagy

**Figure 1:**
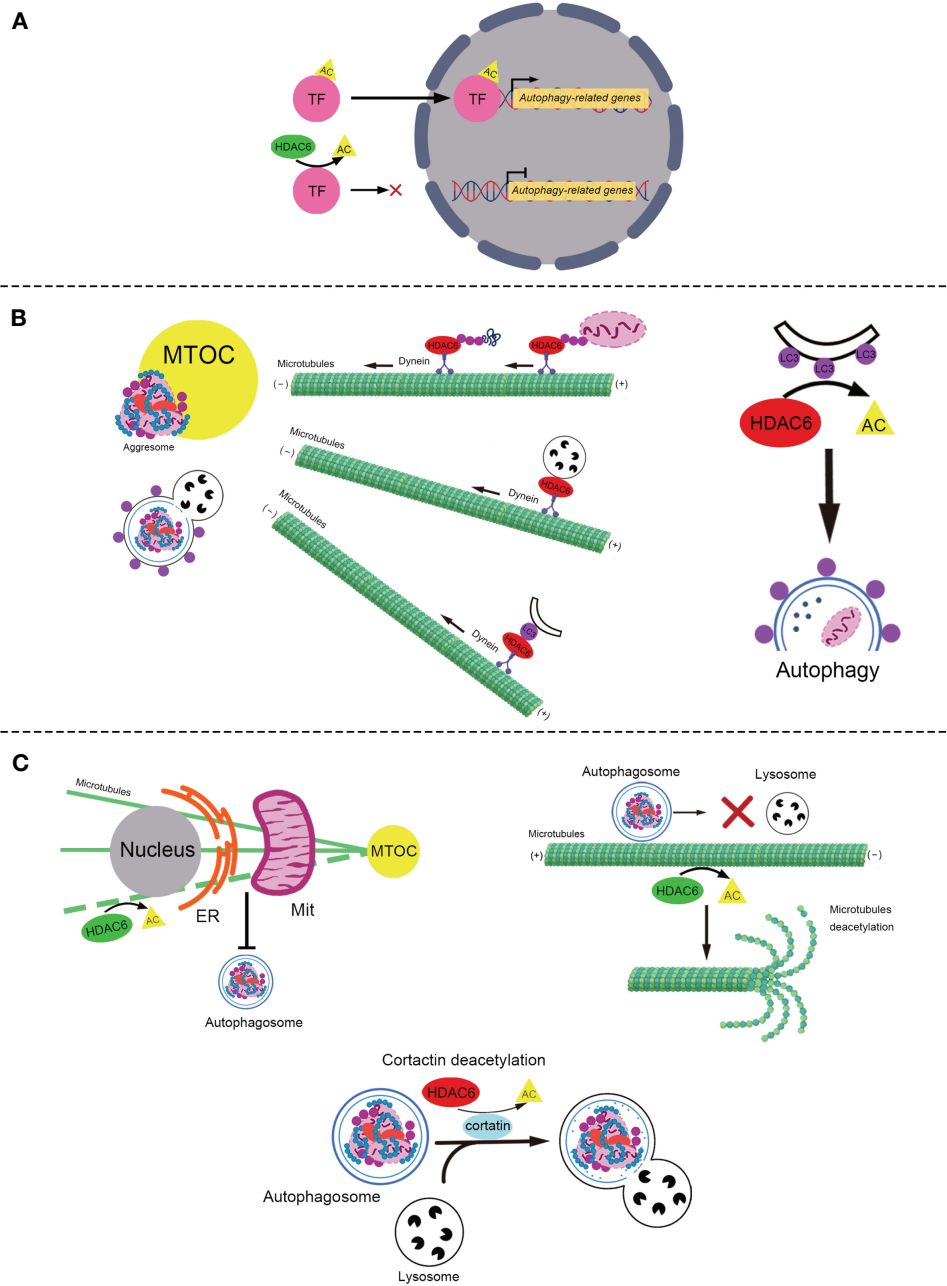
The role of HDAC6 in autophagy. **(A)** The role of HDAC6 in PTM of autophagy-related transcription factors. HDAC6 deacetylates transcription factors, TFEB and FOXO1, to reduce their transcriptional activity and inhibit autophagy. **(B)** HDAC6 promotes the autophagic degradation of aggresome in various ways. Left: HDAC6 interacts with the microtubule motor protein dynein to escort the ubiquitinated misfolded protein or ubiquitinated damaged mitochondria to form the aggresome, to transport the lysosome for the degardaion of aggresome, and to deliver LC3-II (the purple point) to promote the formation of the autophagosome containing aggresome. Right: HDAC6 deacetylates LC3-II to promote the formation of the autophagosome. **(C)** HDAC6 plays various roles in the regulation of autophagy *via* deacetylating α-tubulin and cortactin (positive and negative roles are marked with red and green respectively). Top-left: HDAC6 deacetylates microtubules to block the ER–Mit contact where autophagosome generates. Top-right: HDAC6 suppresses the transport of autophagosomes through deacetylating and reducing the stability of the microtubules. Bottom: HDAC6 blocks the fusion of the autophagosome that contains the misfolded protein or mitochondria and the lysosome by deacetylating cortactin. HDAC6, Histone deacetylase 6; TF, Transcription factor; PTM, Post-translational modifications; AC, Acetylation; FOXO1, Forkhead Box 1; TFEB, Transcription factor EB; MTOC, Microtubule-organizing center; LC3, Microtubule-associated protein 1 light chain 3; ER, Endoplasmic Reticulum; Mit, Mitochondria.

Autophagy, specifically macroautophagy, is a conserved self-eating process that is vital for cellular homeostasis and delivery intracellular components, including soluble proteins, aggregated proteins, organelles, macromolecular complexes, and foreign bodies for degradation (28). This process begins with the sequestration of organelles or portions of the cytoplasm into a double-membrane structure, the autophagosome (29). Autophagosomes fuse with lysosomes to form hybrid organelles called autophagolysosomes (30). Autophagolysosomes degrade the contents to achieve cell homeostasis and organelle renewal (31). HDAC6 is involved in the regulation of autophagy at multiple levels, including participation in post-translational modifications (PTM) of autophagy-related transcription factors (32, 33), the formation of aggresomes that are routinely cleaned through the autophagy pathway (22, 34, 35), and the transportation and degradation of autophagosomes (**Figure 1**) (23, 25).

### The Role of HDAC6 in PTM of Autophagy-Related Transcription Factors

PTM of autophagy-related transcription factors (36), such as transcription factor EB (TFEB) and Forkhead Box 1 (FOXO1) affect their activities, which regulate the autophagy-lysosome pathway (37–39). Recently, it was reported that HDAC6 deacetylates TFEB and FOXO1 to decrease their activity and inhibit autophagy (32, 33, 40, 41).

TFEB is a major regulator of the autophagy-lysosomal pathway (42). Acetylation of TFEB causes translocation to the nucleus and enhancement of autophagy and lysosomal gene transcription (32, 40). It was reported that acetylated TFEB accumulates in the nuclei, which is associated with increased transcriptional activity and lysosomal function following treatment with a pan-HDAC inhibitor, SAHA (40). Similarly, in subtotally nephrectomized rats, the HDAC6 inhibitor Tubastatin A (Tub-A) promotes the acetylation of TFEB, which translocates into the nucleus and enhances the expression of autophagy-related protein Beclin 1 (32), a known direct target of TFEB (43). However, Jung et at. showed that HDAC6 overexpression activated c-Jun NH2-terminal kinase (JNK) and increased the phosphorylation of c-Jun, which activated Beclin 1 dependent autophagy in liver cancer (44).

Besides TFEB, HDAC6 also deacetylates the transcription factor FOXO1 (33), which is a conserved transcription factor that modulates autophagy (45). It has been reported that HDAC6 binds to and deacetylates cytosolic FOXO1, which is required for nuclear translocation and stabilization of interleukin-17 (IL-17)-producing helper T cells (46). Zhang et al. found that trichostatin A (TSA), an HDAC inhibitor, enhances the transcriptional activity of FOXO1 by increasing its acetylation, which enhances the process of autophagy (41). Recently, another study reported that HDAC6 was suppressed by the calcium binding protein S100 calcium binding protein A11 (S100A11) in hepatocytes, which leads to the upregulation of FOXO1 acetylation to enhance its transactional activity and activate autophagy (33).

### The Role of HDAC6 in Aggresome Degradation Mediated by Autophagy

Under physiological conditions, misfolded and aggregated proteins are cleaned through ubiquitylation and proteasome-mediated degradation (47, 48). When the degrading capacity is overwhelmed (47), misfolded or aggregated proteins are generally transported along microtubules towards the microtubule-organizing center (MTOC) through motor protein dynein (49). Once at the MTOC, they are packaged into a single aggresome (49), which is eventually degraded by autophagy (50). Aggresomes are crucial for the clearance of accumulated misfolded proteins and cellular death (51). HDAC6 is a component of aggresomes induced by misfolded proteins. In the process of forming aggresomes containing ubiquitinated proteins, HDAC6 works as a bridge between ubiquitinated-misfolded proteins and the dynein motor (22). It binds to polyubiquitinated misfolded CFTR-ΔF508 *via* its C-terminus ubiquitin binding ZnF-BUZ domain, and it binds to the dynein motor through a separate domain, dynein motor domain (DMB) (22). However, HDAC6 may not recognize protein aggregates and may not bind directly to polyubiquitinated proteins. A recent study indicated that the ZnF-UBP domain of HDAC6 binds to unconjugated C-terminal diglycine motifs of ubiquitin, and this interaction is important for the binding and transport of polyubiquitinated protein aggregates (35). In addition, small-molecule inhibition of HDAC6 has been shown to inhibit the formation of aggresomes in multiple myeloma and lymphoma models (52–54). Recently, HDAC6 was found to be involved in the formation of aggresomes of α-synuclein, TAR DNA-binding protein 43, and Tau (34, 55, 56). It has been suggested that HDAC6 acts as a scaffold for a variety of ubiquitinated proteins. Strikingly, although HDAC6 was initially concentrated at the aggresome as previously reported (22), it was no longer detectable in the ubiquitin-positive structures once aggresomes were cleared by autophagy (57). As the HDAC6 protein levels remained stable during the biological process of aggresome formation and clearance, HDAC6 is not degraded together with aggresomes (57). HDAC6 seems recycled during aggresome-autophagy.

Other studies have shown that HDAC6 is required for lysosomes to form aggregates. Lysosomes are generated in the cell periphery and transported to MTOC to degrade aggresomes (58). HDAC6 and dynein transport lysosomes along microtubules to promote autophagic degradation of aggresomes (59, 60). Lee et al. found that lysosomes in HDAC6 knockout mouse embryonic fibroblasts were dispersed to the cell periphery and not concentrated to protein aggregates (59). Similarly, Iwata et al. also showed that HDAC6 knockdown leads to the periplasmic dispersion of lysosomes (60). This indicates that the targeting of lysosomes to autophagic substrates is regulated by HDAC6.

Microtubule-associated protein 1 light chain 3 (LC3) is a well-known regulator of autophagy (61). LC3-I is conjugated to phosphatidylethanolamine to form LC3-PE conjugate (LC3-II), which is recruited to autophagosomal membranes to promote its formation (62, 63). HDAC6 transports LC3 to the MTOC to promote autophagosome formation (60). The knockdown of HDAC6 attenuates the recruitment of LC3 to aggregated Huntingtin protein for degradation in Neuro2a cells and HeLa cells (60). However, the mechanism by which HDAC6 regulates LC3 needs to be further elucidated. In addition, the deacetylation of LC3 influences autophagy in starvation-induced cells (64). Liu et al. reported that the deacetylation of LC3-II modulated by HDAC6 promotes autophagic flux in starvation-induced HeLa cells (65). The acetylation of LC3-II increases in HDAC6 siRNA Hela cells, which blocks autophagy flux (65). These studies suggested HDAC6 works as a scaffold protein or deacetylase to regulate LC3, which promotes autophagy.

### HDAC6 Deacetylates α-Tubulin and Cortactin to Mediate Autophagy

HDAC6 associates with microtubules and filamentous actin (F-actin) by deacetylating α-tubulin (66–68), and cortactin (19), both of which play important roles in autophagy (69–71). As the first reported and most studied physiological substrate of HADC6, α-tubulin is deacetylated by HDAC6 at lysine 40 (72). Additionally, acetylation of cortactin following inhibition of HDAC6 reduces its interaction with F-actin (19).

Microtubules, composed of α- and β-tubulin heterodimers (73), are essential for cell division, shaping, motility, and intracellular transport (74). Accumulating evidence indicates that microtubules participate in the mediation of autophagosome formation (75, 76), autophagosome transport across the cytoplasm (77, 78), and the formation of autolysosomes (79, 80). Lei et al. demonstrated that HDAC6 decreases the acetylation of microtubules to inhibit the formation of autophagosomes in acidic pH-mediated rat cardiomyocytes (81). The possible underlying mechanism is that acetylation of α-tubulin enhances the endoplasmic reticulum-mitochondria contact, which promotes the formation of autophagosomes (82, 83). Additionally, other studies have reported that HDAC6 mediates α-tubulin deacetylation to suppress autophagy in podocytes and human embryonic kidney 293 cells (84, 85). However, the underlying mechanisms remain unclear. It has been suggested that HDAC6 impairs stable acetylated microtubules *via* deacetylating α-tubulin, which leads to the blockade of autophagosome-lysosome fusion and accumulation of autophagosomes (86). In mouse embryonic fibroblasts, bpV(phen), an insulin mimic and a PTEN inhibitor, blocked autophagosomal degradation by reducing the stability of p62 to activate HDAC6 to impair the fusion of autophagosomes and lysosomes, followed by acetylation of microtubules (86). Furthermore, Li et at. found that HDAC6 inhibited the transportation of autophagosomes to fuse with lysosomes through the deacetylation of α-tubulin, resulting in the depolymerization of microtubules (25). In conclusion, HDAC6 suppresses the formation and degradation of autophagosome *via* deacetylation the microtubules.

As an important part of the cytoskeleton, the F-actin network plays an important role in cell movement, adhesion, morphology, and intracellular material transport (87). Additionally, the F-actin network is essential for the fusion of autophagosomes and lysosomes (70). Lee et al. found that HDAC6 promotes autophagy by recruiting a cortactin-dependent, actin-remodeling machinery, which in turn assembles an F-actin network that stimulates autophagosome-lysosome fusion and substrate degradation (23). However, this mechanism has been demonstrated in quality control autophagy but not in starvation-induced autophagy (23). It is possible that substrates of starvation-induced autophagy are widely distributed in the cell and encounter lysosomes more easily (23). Recently, another study reported that HDAC6 was recruited by ATP13A2, whose mutations are associated with Kufor-Rakeb syndrome (KRS), an autosomal recessive form of juvenile-onset atypical Parkinson’s disease (PD), which is known as Parkinson’s disease-9, to deacetylate cortactin and promote autophagosome-lysosome fusion and autophagy (88). Impaired ATP13A2/HDAC6/cortactin signaling likely contributes to KRS and PD pathogenesis by disrupting the clearance of protein aggregates and damaged mitochondria (88). These results indicate HDAC6 deacetylates cortactin which enhances the activity of the F-actin network to promote the fusion of autophagosomes and lysosomes.

### The Role of HDAC6 in Mitophagy

Mitophagy is an autophagic response that specifically targets damaged and potentially cytotoxic mitochondria (89, 90). HDAC6 has also been reported to mediate mitophagy (88, 91, 92). The underlying mechanisms may include the formation of mitochondrial aggregates (mito-aggresomes) (91–94), and degradation of mitophagosomes through cortactin or α-tubulin action (88, 92, 93, 95). Parkin, a ubiquitin ligase, promotes mitophagy by catalyzing mitochondrial ubiquitination, which in turn recruits ubiquitin-binding autophagic components, HDAC6 and p62, leading to mitochondrial clearance (91, 92). Similar to the aggresome, the formation of mito-aggresomes depends on the transportation of microtubule dynein motors mediated by HDAC6 to MTOC (91, 92). HDAC6 deacetylates cortactin to promote the fusion of mitophagosomes and lysosomes (91, 93). Mito-aggresomes are then degraded by the conventional autophagy pathway (88, 91, 93). Conversely, Pedro et al. found that pharmacological inhibition of the HDAC6 deacetylase activity with Tub-A, did not block striatal neuronal autophagosome-lysosome fusion, suggesting no impairment in mitophagy (95). Interestingly, that HDAC6 inhibition increased acetylated α-tubulin levels, and induced mitophagy in striatal neurons (95). Overall, the effects and mechanisms of HDAC6 in mitophagy remain to be elucidated.

### The Relationship of HDAC6 and p62 in Autophagy

P62 is the first selective autophagy adaptor protein discovered in mammals (96, 97), and plays multiple roles in autophagy, including participating in the formation of aggresomes (98, 99), anchoring the aggresomes to the autophagosome (100), and the degradation of aggresomes in selective autophagy (101, 102). Accumulating evidence indicates that the interaction between HDAC6 and p62 is curial for autophagy (23, 39, 86, 91, 103–108). As mentioned above, HDAC6 and p62 work as two ubiquitin-binding proteins required for efficient autophagy that target protein aggregates and damaged mitochondria (23, 91). Cyclin-dependent kinase 1 (CDK1) in human breast cancer is degraded by p62- and HDAC6- mediated selective autophagy (104). Additionally, interferon-stimulated gene 15 (ISG15) interacts with HDAC6 and p62 independently to be degraded through autophagy (105). These studies suggest that HDAC6 and p62 may mediate autophagy in parallel. However, other studies have indicated that HDAC6 and p62 may regulate autophagy synergistically. Yan et al. reported that HDAC6 regulates lipid droplet turnover in response to nutrient deprivation *via* p62-mediated aggresome formation (107). Interestingly, some studies have indicated that p62 inhibits the deacetylase activity of HDAC6 to enhance the acetylation of microtubules or cortactin, promoting autophagic flux (86, 103, 108). In contrast, Jiang et al. showed that p62 promotes the expression of HDAC6, reducing the acetylation level of microtubules and inhibiting autophagy in hormone-independent prostate adenocarcinoma cell lines (109). However, the mechanisms by which p62 regulates HDAC6 remain to be clarified. The relationship between HDAC6 and p62 is complicated. Thus, further research is required to elucidate the underlying mechanisms.

It is interesting that HDAC6 differentially regulates autophagy *via* multiple mechanisms. It may depend on the specific cell type, disease, and autophagy inducer/inhibitor. The mechanisms of HDAC6 regulation in autophagy require further investigation.

## The Role of HDAC6 in NLRP3 Inflammasome

**Figure 2:**
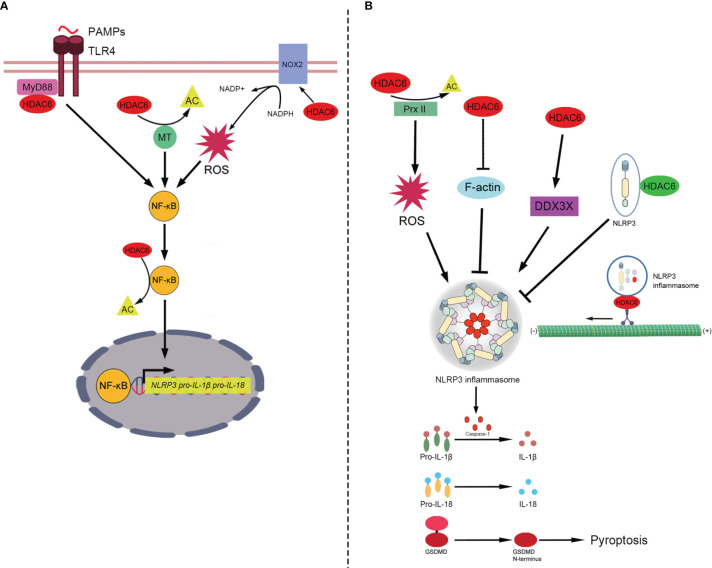
The role of HDAC6 in NLRP3 inflammasome. **(A)** In the priming of NLRP3 inflammasome, HDAC6 promotes NF-κB to enhance the transcription of NLRP3, pro-IL-1β and pro-IL-18. HDAC6 promotes NF-κB in a number of mechanisms. (1) TLR4 senses PAMPs and recruits the downstream adapter proteins MyD88. HDAC6 interacts with MyD88 to enhance the activation of NF-κB. (2) HDAC6 deacetylates microtubules to promote the activity of NF-κB. (3) HDAC6 elevates the expression of NOX2, the component of NADPH oxidase, to promote the level of ROS which upregulates NF-κB activity. (4) HDAC6 directly deacetylates NF-κB. Then, NF-κB upregulates the transcription of NLRP3, pro-IL-1β, and pro-IL-18. **(B)** The role of HDAC6 in the activation and PTM of NLRP3 inflammasome includes a variety of signaling mechanisms (positive and negative roles are marked with red and green respectively). HDAC6 regulates the activation of NLRP3 inflammasome in different ways. (1) HDAC6 suppresses the activity of Prx II *via* deacetylation and increase the level of ROS which is vital for the activation of NLRP3 inflammasome. (2) HDAC6 promotes the activation of NLRP3 inflammasome *via* suppressing F-actin, a negative factor of NLRP3 assembly. (3) HDAC6 enhances the expression of DDX3X. And DDX3X facilitates NLRP3 assembly. In addition, HDAC6 plays both the negative and positive roles in the PTM of NLRP3 inflammasome. The negative one: HDAC6 interacts with ubquitinated NLRP3 protein directly to prevents the activation of NLRP3 inflammasome. The positive one: In an aggresome-like way, HDAC6 works as a dynein adapter to facilitate retrograde transport of NLRP3 inflammasome for activation. Finally, NLRP3 inflammasome releases active caspase-1, which can promote pro-IL-1β/IL-18 to IL-1β/IL-18 and cleave GSDMD to induce pyroptosis. HDAC6, Histone deacetylase 6; NF-κB, Nuclear factor-kappaB; NLRP3, NACHT, LRR, and PYD domains-containing protein 3; Pro-IL-1β, Pro-interleukin-1β; Pro-IL-18, Pro-interleukin-18; PAMPs, Pathogen-associated molecular patterns; TLR4, Toll-like-receptor 4; MyD88, Myeloid differentiation primary response protein 88; AC, Acetylation; MT, microtubule; NADPH, nicotinamide adenine dinucleotide phosphate; NOX2, NADPH oxidase 2; ROS, Reactive oxygen species; Prx II, Peroxiredoxin II; DDX3X, DEAD-Box Helicase 3 X-Linked; F-actin, Filamentous actin; GSDMD, Gasdermin D.

The canonical NLRP3 inflammasome consists of NLRP3 (the sensors), apoptosis-associated speck-like protein containing a caspase recruitment domain (ASC) (the adaptor), and protein-caspase-1 (the effector) (4). It is critical for the innate immune system to mediate caspase-1 activation to release proinflammatory cytokines IL-1β/IL-18 and cleave Gasdermin D to induce pyroptosis in response to microbial infection and cellular damage (110–112). The mechanism of the canonical NLRP3 inflammasome is currently considered to include the following: priming, activation, and PTM- interacting components. The primary signal induces the activation of Toll-like-receptors (TLRs) and nuclear factor-kappa B (NF-κB), leading to transcriptional upregulation of NLRP3, pro-IL-1β, and pro-IL-18 (112). The secondary signal is provided by multiple molecular or cellular events, including ionic flux, mitochondrial dysfunction, and reactive oxygen species (ROS) generation (113). The aberrant activation of NLRP3 inflammasome is responsible for a wide range of inflammatory diseases such as sepsis, trauma and gout (3, 114–116). HDAC6 plays various roles in the priming, activation and PTM of NLRP3 inflammasome (**Figure 2**) (14, 26, 27, 117).

### The Role of HDAC6 in the Priming of NLRP3 Inflammasome

NF-κB, activated by the primary signal, promotes the transcription of NLRP3, pro-IL-1β, and pro-IL-18 (112). The NF-κB transcription factor complex plays a central role in regulating the inducible expression of inflammatory genes in response to immune and inflammatory stimuli. Acetylation of p65, a subunit of NF-κB, has been found to regulate its translocation (118, 119). Jia et al. found that HDAC6 inhibition induces the acetylation of p65 to inhibit its nuclear translocation in diffuse large B-cell lymphoma (120). Xu et al. showed that HDAC6 inhibition upregulated p65 expression in the cytoplasm and reduced p65 expression in the macrophage nucleus to attenuate the transcription of NLRP3 and reduce pyroptosis (27). The inhibition of HDAC6 also reduces p65 expression levels in the nucleus after high glucose stimulation of human retinal pigment epithelium cells, thereby inhibiting the expression of NLRP3 protein and attenuating inflammation (121). These studies suggest HDAC6 deacetylates p65 to upregulate the priming of NLRP3 inflammasome.

Additionally, HDAC6 has been reported to promote the expression of NF-κB to enhance the transcription of pro-IL-1β, increase the release of IL-1β, and aggravate inflammation *via* the interaction of upstream activators of NF-κB, including myeloid differentiation primary response protein 88 (Myd88), α-tubulin, and ROS (93, 122, 123). Gonzalo et al. found that HDAC6 interacts with the TLR adaptor molecule Myd88 (93). The absence of HDAC6 appears to diminish NF-κB induction by TLR4 stimulation and decrease the release of inflammatory factors, including IL-1β (93). Inhibition of HDAC6 upregulates the acetylation of α-tubulin, which decreases the depolymerization of microtubules, to attenuate the activation of NF-κB by blocking IκBα phosphorylation and IL-1β release in mouse lung tissues challenged with lipopolysaccharide (LPS) (122). ROS are mainly produced by NADPH oxidases (124, 125), which are composed of two membrane-bound subunits (p22phox and gp91phox/Nox2), three cytosolic subunits (p67phox, p47phox, and p40phox), and a small G-protein Rac (Rac1 and Rac2) (126). HDAC6 upregulates the expression of Nox2-based NADPH oxidase subunits to increase the production of ROS (123, 127–129), which promotes NF-κB activation and IL-1β release (123, 127). Given that the maturation and release of pro-IL-1β are mainly mediated through inflammasome-activating caspase-1 (130, 131), it is possible that HDAC6 stimulates NF-κB activation *via* Myd88, microtubules or ROS to activate NLRP3 inflammasomes. However, the underlying mechanisms remain to be elucidated.

### The Role of HDAC6 in the Activation of NLRP3 Inflammasome

Following the primary signal that licenses the cell, the secondary signal occurs following the recognition of an NLRP3 activator and induces full activation and inflammasome formation (113). NLRP3 is activated by a wide variety of stimuli including ROS (132–134). The crystal structure of NLRP3 contains a highly conserved disulfide bond connecting the PYD domain and the nucleotide-binding site domain, which is highly sensitive to altered redox states (135). Redox regulatory proteins, Prx I and Prx II, are highly homologous 2-cysteine members of the Prx protein family that function as antioxidants at low resting levels of H2O2, an ROS (136). Prx I and Prx II are specific targets of HDAC6 deacetylases. Inhibition of HDAC6 increases the levels of acetylated Prx I and Prx II (20, 137). Recently, Yan et al. reported that pharmacological inhibition of HDAC6 attenuates the expression of NLRP3 and mature caspase-1 and IL-1β, and protects dopaminergic neurons *via* Prx II acetylation, which reduces ROS production (26). These studies suggest that HDAC6 also mediates the activation of NLRP3 inflammasome, probably through Prx I and Prx II deacetylation which upregulates ROS production. However, with the treatment of LPS, ZnF-BUZ but not deacetylase domains facilitates the activation of NLRP3 inflammasome in mouse bone marrow-derived macrophages (iBMDM) (14). Hence, the role of deacetylase domains in the activation of NLRP3 inflammasome remain to be elucidated.

Additionally, HDAC6 inhibitor ACY1215 downregulates the activation of NLRP3 inflammasome *via* modulating F-actin and DEAD-Box Helicase 3 X-Linked (DDX3X) (138, 139). F-actin acts as a negative regulator by interacting directly with NLRP3 and ASC, following the activation of NLRP3 inflammasome (140). Flightless-I (FliI) and leucine-rich repeat FliI-interaction protein 2 (LRRFIP2) are required for the co-localization of NLRP3, ASC, and F-actin (140). Recently, Chen et al. reported that the HDAC6 inhibitor ACY1215 decreases the activation of NLRP3 inflammasome in acute liver failure (ALF) by increasing the expression of F-actin (138). However, the mechanism underlying HDAC6 inhibition that upregulates the expression of F-actin still needs to be elucidated. Interestingly, another study also found similar results that ACY1215 inhibits the activation of M1 macrophages by regulating NLRP3 inflammasome in ALF, but by a different mechanism (141). In LPS-stimulated ALF mice, ACY1215 decreased the expression of NLRP3 and increased the expression of DEAD-Box Helicase 3 X-Linked (DDX3X) (141), a critical factor for NLRP3 inflammasome assembly (139). It is suggested that the DDX3X/NLRP3 pathway is involved in the protective effects of the HDAC6 inhibitor ALF, but the interaction of HDAC6 and DDX3X needs to be further studied.

### The Role of HDAC6 in the PTM of NLRP3 Inflammasome

PTM, including ubiquitination, deubiquitination, phosphorylation, and degradation, occurs in almost every aspect of inflammasome activity, and can either lead to the activation of the inflammasome or suppression of inflammasome activation (142). Recently, Magupalli et al. proved that NLRP3 inflammasome activation depends on regulated ubiquitination (143, 144) and engagement of the dynein adaptor HDAC6 to transport NLRP3 inflammasome to the MTOC for activation in a ubiquitin-misfolded protein-like manner (14). However, it is unknown which inflammasome components need to be ubiquitinated. Hwang et al. previously reported that HDAC6 negatively regulates NLRP3 inflammasome activation through its interaction with ubiquitinated NLRP3 (117). Co-immunoprecipitation data revealed a specific association between HDAC6 and NLRP3 (117). PR619 treatment (deubiquitinase inhibitor) resulted in an increase in the interaction of NLRP3 with HDAC6 and a decrease in NLRP3-dependent caspase-1 activation (117). This indicates that the Zn-BUZ domain of HDAC6 might interact with ubiquitinated NLRP3 (117). The effect of HDAC6 on the PTM of NLRP3 inflammasome is controversial, although previous studies indicated that the HDAC6 ubiquitin-binding domain but not deacetylase activity, is required for NLRP3 activation.

## Discussion

**Figure 3:**
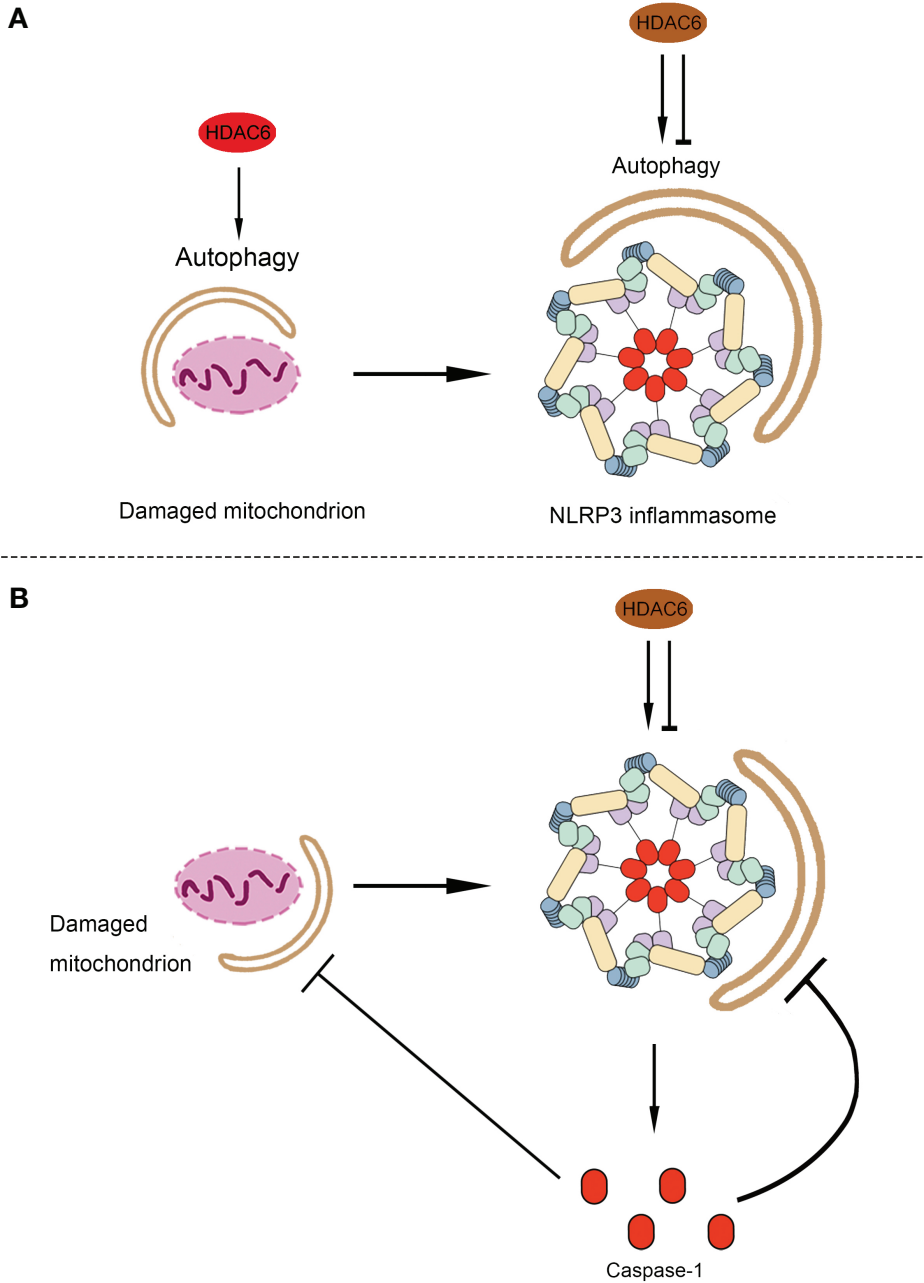
The possible role of HDAC6 in the crosstalk between autophagy and NLRP3 inflammasome. **(A)** The possible role of HDAC6 in the autophagic degradation of the component of NLRP3 inflammasomes and endogenous inflammasome activators. As a source of endogenous inflammasome activators, damaged mitochondrion promotes the activation of NLRP3 inflammasome. Both the damaged mitochondrion and the component of NLRP3 inflammasome can be limited by autophagy. HDAC6 may promote the mitophagy and then inhibit the activation of NLRP3 inflammasome indirectly. Moreover, it is possible that the HDAC6 upregulate or downregulate autophagy to affect NLRP3 inflammasome. **(B)** The possible role of HDAC6 in the regulation of autophagy by NLRP3 inflammasome. HDAC6 plays a dual role in the activation of NLRP3 inflammasome, which release the caspase-1. Caspase-1 inhibits the autophagic degradation of the damaged mitochondrion or the component of NLRP3 inflammasome. Hence, HDAC6 may regulate autophagy *via* the activation of NLRP3 inflammasome. HDAC6, Histone deacetylase 6; NLRP3, NACHT, LRR, and PYD domain-containing protein 3.

The association between autophagy and inflammasomes was discovered more than ten years ago. Satioh et al. first reported the interplay between autophagy and the endotoxin-induced inflammatory immune response through activation of the inflammasome and release of cytokines (145). In LPS-stimulated macrophages, autophagy-related protein Atg16L1 (autophagy-related 16-like 1) deficiency resulted in increased caspase-1 activation, leading to increased IL-1β production (145). Since then, Nakahira *et al.* indicated that autophagic proteins regulate NLRP3-dependent inflammation by preserving mitochondrial integrity (146). LC3B-deficient mice produced more caspase-1-dependent cytokines in sepsis models and were susceptible to LPS-induced mortality than controls (146). In the last decade, numerous studies have further indicated that autophagy can affect NLRP3 inflammasome activation through various mechanisms (147). Autophagy can suppress NLRP3 inflammasome activation by removing endogenous inflammasome activators, such as ROS-producing damaged mitochondria (148) and removing inflammasome components (149) and cytokines (150). Additionally, NLRP3 inflammasome activation regulates autophagosome formation through various mechanisms. Silencing NLRP3 downregulated autophagy (151, 152). Interestingly, caspase-1 also regulates the autophagic process through cleavage of other substrates (153, 154). Interplay between autophagy and NLRP3 inflammasomes is essential for the balance between the required host defense inflammatory response and prevention of excessive inflammation. As mentioned above, previous studies have shown that HDAC6 mediates the process of autophagy and the functioning of NLRP3 inflammasomes *via* multiple mechanisms. However, the role of HDAC6 in the crosstalk between autophagy and NLRP3 inflammasome is poorly understood. In the following sections, we will discuss the possible link between HDAC6 and the interplay between autophagy and inflammasomes, considering the current evidence (**Figure 3**).

### The Possible Role of HDAC6 in the Autophagic Degradation of the Component of NLRP3 Inflammasomes and Endogenous Inflammasome Activators

Components of NLRP3 inflammasome, including NLRP3 and ASC, are recognized by p62, a ubiquitin-binding protein, that forms aggresomes and is degraded by autophagy (149). Similarly, a recent study by Han et al. showed that small molecules (kaempferol-Ka) induced autophagy to promote the degradation of inflammasome components and reduce inflammasome activation in an LPS-induced Parkinson disease mouse model (155). As described previously, HDAC6 can also function as a ubiquitin-binding protein to participate in aggresome formation (22). Additionally, HDAC6 can also mediate the acetylation of cortactin and microtubules to regulate autophagy *via* autophagosome-lysosome fusion and autophagosome transportation (23, 86, 156). Furthermore, HDAC6 interacts with p62 to regulate autophagy *via* various mechanisms (23, 91, 104, 107). Although, there are no studies indicating that HDAC6 promotes autophagy to reduce the activation of NLRP3 inflammasomes directly, according to the current evidence, it is possible that HDAC6 participates in the autophagic degradation of the components of NLRP3 inflammasomes to regulate its activation.

On the other hand, autophagy removes damaged organelles, such as mitochondria, leading to a reduction in the release of mitochondrial-derived damage-associated molecular patterns (DAMPs), mitochondrial ROS (mtROS), and mitochondrial DNA (mtDNA) (148, 157). Numerous studies have shown that Parkin-mediated mitochondrial autophagy suppresses the production of mtROS and mtDNA, which inhibits the activation of NLRP3 inflammasomes (158–162). As mentioned above, following the decoration of mitochondria with ubiquitin by Parkin, HDAC6 is recruited as a ubiquitin-binding autophagic component that causes mitochondrial clearance (91–93). This evidence suggests that HDAC6 may mediate the functioning of NLRP3 inflammasome *via* mitophagy eliminating mtROS and mtDNA.

### The Possible Role of HDAC6 in the Regulation of Autophagy by NLRP3 Inflammasome

Following the activation of the NLPR3 inflammasome, caspase-1 cleaves some components of autophagy to block this process (153, 154). Yu et al. showed that caspase-1 triggers mitochondrial damage *via* cleavage of Parkin inhibiting mitophagy, following its activation by NLRP3 and melanoma 2 (AIM2) inflammasomes (153). Furthermore, caspase-1 mediated cleavage of the signaling intermediate Toll-interleukin-1 receptor (TIR)-domain-containing adaptor-inducing interferon-β (TRIF), an essential part of the TLR4-mediated signaling pathway, leading to the promotion of autophagy (154). As HDAC6 regulates the priming, activation, and PTM of NLRP3 inflammasome (14, 26, 27, 117), it is possible that HDAC6 may regulate autophagy through the activation of NLRP3 inflammasomes. The regulation of the crosstalk between autophagy and NLRP3 inflammasome machinery by HDAC6 is obviously complex and requires further investigation, and may be dependent on specific conditions such as cell type, model of disease, inflammasome activator, and autophagy inducer/inhibitor.

## Conclusion and Perspective

An increasing number of studies have reported the crosstalk between NLRP3 inflammasome and autophagy in various models and diseases in the last ten years. Numerous studies have indicated that autophagy suppresses NLRP3 inflammasome activation, through various mechanisms. In addition, NLRP3 inflammasome activation regulates autophagosome formation *via* multiple mechanisms. The crosstalk between autophagy and NLRP3 inflammasome is essential for host defense and the inflammatory response. On the other hand, accumulating evidence indicates that HDAC6 plays important roles in the mediation of autophagy and functioning of NLRP3 inflammasome *via* differential mechanisms. However, the role of HDAC6 in the crosstalk between autophagy and NLRP3 inflammasome remains poorly understood. In this review, we explored the possible link between HDAC6 and the interplay between autophagy and inflammasomes, considering the current evidence. HDAC6 is a promising therapeutic target in multiple diseases including inflammatory diseases, cancer, and autoimmune diseases. With the development of small molecules inhibiting HDAC6, some clinical trials have shown that selective HDAC6 inhibitors are effective in tumor treatment (163–166). It is worth noting that the effects of HDAC6 differ in specific cell types and conditions. Considering the role of HDAC6 in autophagy and NLRP3 inflammasome, HDAC6 inhibitors have broad prospects and should be studied further deserves to pursue in future research.

## Author Contributions

Conception and design – PC and TW. Manuscript preparation – PC and HL. Critical revisions – PC, HL, HH, YL, and TW. All authors contributed to the article and approved the submitted version.

## Funding

This work is supported by National Natural Science Foundation of China, 82102315 (PC), Beijing Natural Science Foundation, 7214265 (PC), National Natural Science Foundation of China, 31771326 (TW), and UMHS-PUHSC Joint Institute for Translational and Clinical Research, BMU2020JI007 (TW).

## Conflict of Interest

The authors declare that the research was conducted in the absence of any commercial or financial relationships that could be construed as a potential conflict of interest.

## Publisher’s Note

All claims expressed in this article are solely those of the authors and do not necessarily represent those of their affiliated organizations, or those of the publisher, the editors and the reviewers. Any product that may be evaluated in this article, or claim that may be made by its manufacturer, is not guaranteed or endorsed by the publisher.
